# Alternate day fasting on subjective feelings of appetite and body weight for adults with overweight or obesity: a systematic review

**DOI:** 10.1017/jns.2022.90

**Published:** 2022-10-31

**Authors:** Bahar Kucuk, Rigmor C. Berg

**Affiliations:** 1 Department of Community Medicine, The Arctic University of Norway, Tromsø, Norway; 2 Department of Reviews and Health Technology Assessments, The Norwegian Institute of Public Health, Oslo, Norway

**Keywords:** Alternate day fasting, Appetite, Body weight, Fullness, Hunger, Weight loss

## Abstract

Alternate day fasting (ADF) with consumption of calories up to 25 % of the daily energy intake on fast days is one of the most used intermittent fasting regimens and promoted as a promising, alternative approach for treating obesity. Feelings of appetite are critical for adherence to dietary approaches, and therefore the success of dietary interventions. This systematic review aimed to assess the effects of a minimum of 8 weeks of ADF on subjective feelings of appetite and body weight for adults with overweight and obesity. We conducted the review in accordance with the Cochrane guidelines, including systematic searches in four databases. Because of the high level of clinical and methodological heterogeneity, a narrative approach was used to synthesise the results. Eight studies with a total of 456 participants met the eligibility criteria: three randomised controlled trials and five uncontrolled before-after studies. Seven of the studies had high risk of bias. Feelings of appetite were assessed by hunger in eight studies, fullness in seven studies, satisfaction in four studies and desire to eat in one study. All the studies assessed weight loss. The certainty of the evidence was rated low or very low for all outcomes, thus no firm conclusions can be drawn about the potential benefits of ADF on subjective feelings of appetite and body weight. Despite the high interest in ADF, good quality evidence is still needed to determine its effectiveness and if offered in clinical practice, ADF should be offered cautiously while concomitantly evaluated.

## Introduction

Obesity is a worldwide epidemic and a well-known risk factor for many metabolic disorders^(1)^. Over time, obesity results in metabolic changes that are associated with an increased risk for morbidity and mortality as well as reduced life expectancy^(2,3)^. Lifestyle interventions have been documented to improve metabolic abnormalities linked to obesity. An emerging body of evidence shows that adherence to dietary prescriptions, regardless of diet composition, is the strongest predictor of weight loss^(4,5)^. However, with dietary prescriptions, people experience changes in appetite, cravings, diet satisfaction^(4,6)^, as well as a rise and fall of hunger that drives patterns of food intake^(7)^. These changes that ordinarily occur during weight loss likely contribute to poor motivation and dietary compliance. They also point to important ways that the human appetite system is linked to obesity^(4,6)^. With weight loss, feelings of hunger are promoted to restore a normal body weight status. These compensatory changes are thought to be induced by alterations in expression of hypothalamic regulators of energy balance as well as adaptive changes in gut function, which alter the concentration of appetite-regulating hormones, such as ghrelin, cholecystokinin and peptide tyrosine tyrosine (PYY)^(8)^. These are likely some of the reasons that weight loss is difficult to maintain for most people^(7–9)^. Furthermore, the homeostatic control of food intake is strongly influenced by hedonistic impulses, the reward system and eating experiences^(10,11)^.

When applied long term, one strategy thought to suppress appetite, while simultaneously avoiding the compensatory increases in hunger that usually occur during energy restriction, is very-low-calorie diets (VLCDs) ≤3347 kJ/d (800 kcal/d)^(2,8)^. These diets use severe energy restriction, including a low carbohydrate intake, which creates a metabolic response with an increased circulation of ketone bodies produced by the liver, which is also called the metabolic switch^(2,8)^. VLCDs have been shown to be effective in the treatment of obesity. Weight loss following VLCDs was reported in several reviews especially when applied in addition to a behavioural programme^(12–15)^. However, individuals who struggle to cope with sensations of hunger may not maintain sufficient levels of severe restriction to experience the beneficial changes in sensations of satiety that help to increase dietary adherence^(2,13,16)^. VLCDs may also become monotonous and may cause the body to adapt and therefore prevent further weight loss^(17–20)^.

Alternate day fasting (ADF), also called every other day fasting, is an alternative approach to weight loss thought to be easier to follow than VLCD. This is because ADF involves shorter spells of intense energy restriction followed by periods of *ad libitum* intake^(21)^. It involves a 24 h fast day, which allows the consumption of VLCDs up to 25 % of daily calorie needs, alternated with a 24 h feed day during which people usually eat *ad libitum*^(22,23)^. ADF is one of the most popular intermittent energy restriction (IER) approaches in the scientific literature^(24)^. The use of ADF protocols has been mainly aimed at counteracting obesity and maximising the effects on healthy living^(25)^. Many studies have shown that IER, including ADF, has additional benefits from its effects on metabolic switching, such as reversing insulin resistance, strengthening the immune system, improving systemic inflammatory diseases, enhancing physical and cognitive functions, protecting against neurodegeneration and even expanding the life span^(25–27)^. Several systematic reviews on the effects of ADF on weight loss showed that ADF effectively lowered body weight^(28–30)^. For example, a recent review of seven randomised controlled trials (RCTs) conducted by Cui *et al.* reported that compared with the control group, the ADF group showed statistically significant reductions in weight and body mass index.^(30)^ ADF also appears to achieve greater weight loss than other IER approaches^(31,32)^. This may be because ADF increases individuals’ awareness of food habits, and reassures them that they can manage the high levels of hunger on restriction days^(33)^. It may also decrease the monotony, and, because people know that food will not be restricted the next day, increase their motivation^(17)^. Additionally, given ADF only requires a restriction of food intake every other day, it may prevent body adaptations to the diet that hinders further weight loss. Due to the periodic nature of fasting, it may also mitigate the constant hunger that practitioners of continuous or daily energy restriction (CER or DER) endure^(34)^.

Although reviews of ADF exist, a careful examination of the effect of ADF on appetite regulation is missing in the scientific literature. A better understanding of the effect of ADF on subjective measures of appetite is critical to evaluate the potential of this approach as a dietary treatment for individuals with overweight or obesity. The purpose of this review is, therefore, to systematically review the available evidence and summarise the effects of ADF on subjective feelings of appetite as a primary outcome, and weight change as a secondary outcome, for adults with overweight or obesity.

## Methodology

This systematic review was conducted in accordance with the Cochrane Handbook for Systematic Reviews of Interventions^(35)^. The study protocol was registered in PROSPERO in May 2021 (CRD42021247708). We report in line with the PRISMA checklist^(36)^.

### Eligibility criteria

We applied the (S)PICO model, which directs attention to the study design, population, intervention, comparison and outcomes^(37)^. This systematic review considered RCTs, non-RCTs and controlled and uncontrolled before-after (UCBA) studies. Studies with participants aged between 18 and 65 years, who were overweight or obese according to the WHO with a BMI ≥25 or 30 kg/m^2(38)^, respectively, were included. We excluded adults with unstable body weight (understood as >4 kg weight loss or gain 3 months prior to the beginning of the study), patients with chronic infections, cancer or history of eating disorders, individuals who had bariatric surgery, pregnant women, and those planning a pregnancy or breastfeeding. In the event of mixed populations, we considered studies when at least 75 % of the sample fit the inclusion criteria.

With respect to the intervention, studies were eligible if ADF consisted of a cyclical feeding pattern with a fast day characterised by 0–25 % of the individuals' daily energy needs for a period of 24 h, alternated with a feast day of no caloric-restriction or the possibility to eat *ad libitum* for 24 h. In the event of no or limited information about the percentage of the diet, we excluded studies with energy intake of more than 2510 kJ (600 kcal) on fast days. While there is no gold standard for the duration of ADF, 8 weeks is the most common duration found in the literature, thus we included interventions of minimum 8 weeks. CER, DER and *ad libitum* diets (unrestricted) were eligible as the comparator for controlled studies.

The primary outcome of interest was feelings of appetite (hunger, fullness, satisfaction and desire to eat) which had to be assessed by quantitative scales such as numeric rating scales or visual analogue scales (VAS). The secondary outcome was quantitatively described body weight or weight change. Lastly, published journal articles in English, from 2000 to 2021, were eligible. If there were more than one publication based on the same study population, the most informative publication for our purposes was included.

### Search strategy

The main search strategy was systematic searches in four electronic databases, up to February 2021 (full search strategy can be found in Supplementary Appendix 1). To identify studies not indexed in databases, we also searched Google Scholar and the reference lists of all included studies, relevant systematic reviews, literature reviews and other relevant publications.

### Selection of studies

We imported the records identified in the database searches to EndNote X9^(39)^ and removed duplicates. Next, we imported the records to the screening tool Rayyan QCRI, which is a web-tool designed to help researchers working on systematic reviews and other knowledge syntheses^(40)^. We performed study selection in two stages. First, using Rayyan, we screened all titles and abstracts against the inclusion criteria, and in the second step, the full text of studies deemed eligible in the first step.

### Data extraction and risk of bias assessments

We systematically extracted data from the included sources, using a data extraction form, which was adapted from the Cochrane Effective Practice and Organization of Care Group resources^(41)^. The extracted data included: title, authors and other details of the publication; study design, setting and aim; characteristics of the population; characteristics of the intervention and the comparator; details of outcome measurements and results related to the outcomes. For data that were only presented in figures, we used the GetData Graph Digitizer version 2·26^(42)^ to extract data.

We assessed risk of bias in RCTs with the Cochrane Risk of bias tool for RCTs (RoB2)^(35)^. For other study designs, we used the Risk of Bias Assessment Tool for Non-Randomized Studies (RoBANS)^(43)^.

### Data synthesis and assessment of the certainty of evidence

As stated in our protocol, we planned to extract crude outcome data for all eligible outcomes and adjusted outcome data (adjusted comparison estimates and their standard errors or 95 % confidence intervals, CIs). Continuous outcomes would be presented as mean differences (MDs) along with standard deviations, standard error of the means or 95 % CIs and dichotomous outcomes as the number of events and the number of people in groups as proportions, risk ratio (RR), incident RR or odds ratio as appropriate, or the most appropriate presentation based on the available data in the included studies. However, outcome data and effect estimates were often missing and only a significance level was provided. To the extent possible, we extracted data from figures using GetData Graph Digitizer^(42)^ and calculated additional data such as area under curve (AUC) from individual-level data points when possible.

We planned to conduct meta-analyses if the included studies were sufficiently similar, and data were available. Thus, we evaluated the characteristics of the studies’ (S)PICO and based our judgements about whether meta-analyses were appropriate on recommendations in the Cochrane Handbook^(35)^. Cochrane Handbook recommends narrative synthesis for reviews where meta-analysis is either not feasible or not sensible. For example, when data are missing, data are from different study designs or when the studies capture a wide range of PICO^(44)^. Therefore, because there were biases arising from the study designs, clinical and methodological heterogeneity across the studies, and missing and differentially reported outcome data, we adopted a narrative approach to synthesise the results. Furthermore, given fewer than ten studies were included and there was high heterogeneity, we could neither conduct sensitivity and subgroup analyses nor test for funnel plot asymmetry, as planned in the protocol.

The certainty in the evidence was assessed by using the Grading of Recommendations Assessment, Development and Evaluation (GRADE) framework^(45,46)^. We used the recommendations in Murad *et al.*^(46)^ for assessing the certainty of evidence in the absence of a single effect estimate. We used GRADE to create a *summary of findings* table.

## Results

**Fig. 1.**
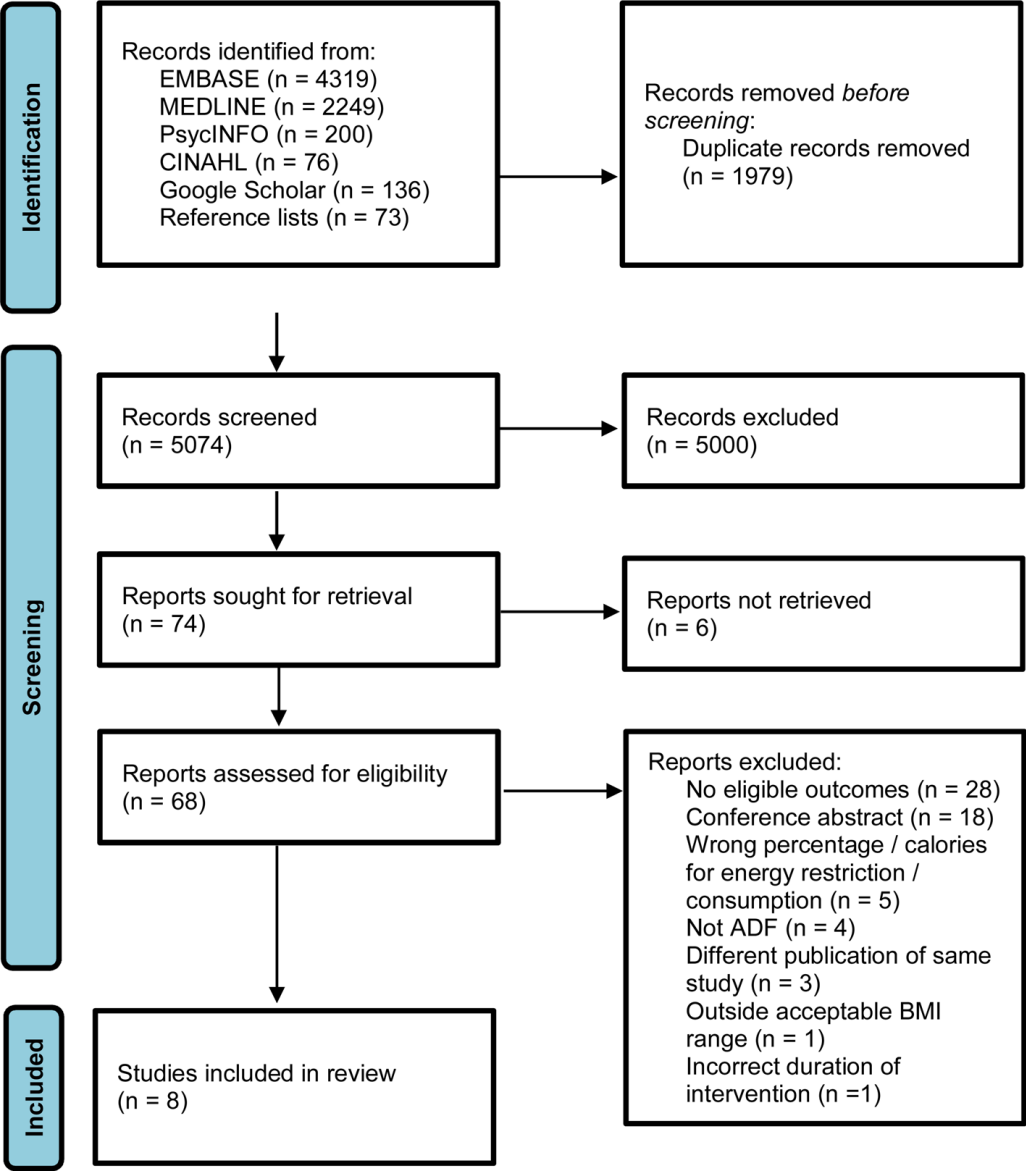
Flow diagram of the literature selection process.

After the removal of duplicates, we screened 5074 records (Fig. 1). Of sixty-eight full texts assessed for eligibility, eight studies met the inclusion criteria. The excluded records read in full text can be found in Supplementary Appendix 2.

### Description of the included studies

The characteristics of the included studies are summarised in Table 1. The included studies were published between 2006 and 2020. Three studies were RCTs and the remaining studies were UCBA studies. One was designed as an RCT^(47)^, but we treated it as a UCBA study with two eligible intervention arms because it lacked an eligible control group: there was a high-fat diet group, consisting of 45 % of energy as fat, and a low-fat diet group, with 25 % of energy as fat. Six studies were from the USA, one was from England and one was from China. Across the studies, there were 456 participants at baseline, of which the majority were women. Two studies consisted of only women^(16,47)^. The age ranges were 18–65 years and the mean age was from 34 to 48 years. The mean BMI was between 26 and 38 kg/m^2^. The studies included participants from multiple ethnic backgrounds.

**Table 1.** Description of the included studies (*N* 8)

Author, Country, Study Design	Population	Intervention and comparator (follow-up time)	Outcomes
Beaulieu *et al.* 2019^(16)^ (England) RCT	Women with overweight and obesity *N* 46 (ADF *n* 24, CER *n* 22) Age: 18–55 years BMI: 25–34·9 kg/m^2^	ADF: 25 % of baseline energy needs on fast days (24 h) alternating with *ad libitum* feed days (24 h) CER: 75 % of baseline energy needs (12 weeks)	Hunger Fullness Desire to eat Body weight
Bhutani *et al.* 2013^(48)^ (USA) RCT	Adults with obesity *N* 41 (ADF *n* 25, Control *n* 16) Age: 25–65 years BMI: 30–39·9 kg/m^2^	ADF: 25 % of baseline energy needs on fast days (24 h) alternating with *ad libitum* feed days (24 h) – 4 weeks controlled feeding period followed by 8 weeks ADF self-selected feeding period Control group: usual food habits (12 weeks)	Hunger Fullness Satisfaction Body weight change
Cai *et al.* 2019^(31)^ (China) RCT	Adults with non-alcoholic fatty liver disease *N* 174 (ADF *n* 95, CER *n* 79) Age: 18–65 years BMI: >24 kg/m^2^	ADF: 25 % of baseline energy needs on fast days (24 h) alternating with *ad libitum* feed days (24 h) CER: 80 % of baseline energy needs (12 weeks)	Hunger Fullness Satisfaction Body weight change
Hoddy *et al.* 2016^(22)^ (USA) UCBA	Adults with obesity N 74 Age: 25–65 years BMI: 30–39·9 kg/m^2^	ADF: 25 % of baseline energy needs on fast days (24 h) alternating with *ad libitum* feed days (24 h)* No control group (8 weeks)	Hunger Fullness Body weight change
Johnson *et al.* 2006^(49)^ (USA) UCBA	Adults with obesity and moderate asthma *N* 14 Age: adults BMI: >30 kg/m^2^	ADF: 13 39 kJ (320 kcal) for women and 1590 kJ (380 kcal) for men on fast days (24 h) alternating with *ad libitum* feed days (24 h) No control group (8 weeks)	Hunger Body weight change
Kalam *et al.* 2020^(50)^ (USA) UCBA	Adults with obesity *N* 52 Age: 18–65 years BMI: 30–50 kg/m^2^	ADF: 25 10 kJ (600 kcal) low carbohydrate diet on fast days (24 h) alternating with 4184 kJ (1000 kcal) + *ad libitum* feed days (24 h) for 12 weeks; then 25 10 kJ (600 kcal) + *ad libitum* feed days (24 h) for 12 weeks No control group (24 weeks)	Hunger Fullness Body weight change
Klempel *et al.* 2013^(47)^ (USA) UCBA	Women with obesity *N* 35 (High-fat diet group *n* 17, Low-fat diet group *n* 18) Age: 25–65 years BMI: 30–39·9 kg/m^2^	ADF: 25 % of baseline energy needs (high-fat or low-fat diet) on fast days (24 h) alternating with *ad libitum* feed days (24 h)* No control group (8 weeks)	Hunger Fullness Satisfaction Body weight change
Klempel *et al.* 2010^(51)^ (USA) UCBA	Adults with obesity *N* 20 Age: 35–65 years BMI: 30–39·9 kg/m^2^	ADF: controlled feeding for 4 weeks then self-selected feeding period for 4 weeks with 25 % of baseline energy needs on fast days (24 h) alternating with *ad libitum* feed days (24 h)* No control group (8 weeks)	Hunger Fullness Satisfaction Body weight change

RCT, randomised controlled trial; ADF, alternate day fasting; BMI, body mass index; CER, continuous energy restriction; h, hours; UCBA, uncontrolled before-after study; kJ, kilojoule; kcal, kilocalorie.

* There was a 2 weeks baseline period, followed by ADF.

With respect to the characteristics of the intervention, six of the studies used an ADF protocol with a consumption of 25 % of baseline energy needs on fast days and two of the studies used calories instead of percentages^(49,50)^. For the feed days, seven of the studies allowed *ad libitum* consumption. The study by Klempel *et al.*^(47)^ used a consumption of 125 % of baseline energy needs on feed days. Fat, carbohydrate and protein composition of diet on fast days differed between studies, being 24–45 %, 30–60 % and 15–37 %, respectively. In all eight studies, food was provided on fast days. Across the studies, the intervention duration was from 8 to 24 weeks. While the five UCBA studies^(22,47,49–51)^ had no comparison groups, two RCTs had CER diet as the control group^(16,31)^ and the control group in the last RCT had no specified diet^(48)^. The details of the ADF intervention across the included studies are shown in Table 2.

**Table 2.** Various components of ADF protocols in the included studies

Study	Beaulieu *et al.* 2019	Cai *et al.* 2019	Bhutani *et al.* 2013	Kalam *et al.* 2020	Hoddy *et al.* 2016	Klempel *et al.* 2013	Klempel *et al.* 2010	Johnson *et al.* 2006
Calorie intake on fast days	25 % of baseline energy needs	25 % of baseline energy needs	25 % of baseline energy needs	25 10 kJ (600 kcal)	25 % of baseline energy needs	25 % of baseline energy needs	25 % of baseline energy needs	13 39 kJ (320 kcal) for women and 1590 kJ (380 kcal) for men
Calorie intake on feed days	*Ad libitum*	*Ad libitum*	*Ad libitum*	4184 kJ (1000 kcal) + *Ad libitum* for the first 3 months 25 10 kJ (600 kcal) + *Ad libitum* for the last 3 months	*Ad libitum*	125 % of baseline energy needs	*Ad libitum*	*Ad libitum*
Macronutrient composition of the diet on fast days	27 % fat, 36 % CHO, 37 % protein	30 % fat, 55 % CHO, 15 % protein	30 % fat, 55 % CHO, 15 % protein	35 % fat, 30 % CHO, 35 % protein	24 % fat, 60 % CHO, 16 % protein	High-fat group: 45 % fat, 40 % CHO, 15 % protein Low-fat group: 25 % fat, 60 % CHO, 15 % protein	Controlled feeding phase 25 % fat, 52 % CHO, 23 % protein	Not stated
Controlled feeding on fast days	✓	✓	✓	✓	✓	✓	✓	✓
Self-selected feeding on fast days			✓				✓	
Specific timing for fast day meals		Between 12·00 and 14·00	Between 12·00 and 14·00			Between 12·00 and 14·00	Between 12·00 and 14·00	
Use of food diaries	✓		✓	✓	✓	✓	✓	✓
Dietary counselling	✓		✓	✓	✓		✓	
Group support meetings								✓

ADF, alternate day fasting; kJ, kilojoule; kcal, kilocalorie; CHO, carbohydrate.

As per our inclusion criteria, all eight studies assessed feelings of appetite. Specifically, all eight studies assessed hunger, seven assessed fullness^(16,22,31,47,48,50,51)^, four assessed satisfaction^(31,47,48,51)^ and one study assessed desire to eat^(16)^. Except for the study by Johnson *et al.*^(49)^ that used a numeric rating scale (from 1 = not at all to 10 = extremely), all studies used VAS for subjective appetite measurements (from 0 mm = not at all to 100 mm = extremely). Only one study stated that a validated electronic system was used for quantification^(16)^. In three studies, appetite rating assessments were measured pre-prandial, immediately after consumption and post-prandial^(16,22,50)^. Therefore, AUC values were available for these studies. Our secondary outcome was body weight and all eight studies examined either body weight or body weight changes.

### Risk of bias of the included studies

**Fig. 2.**
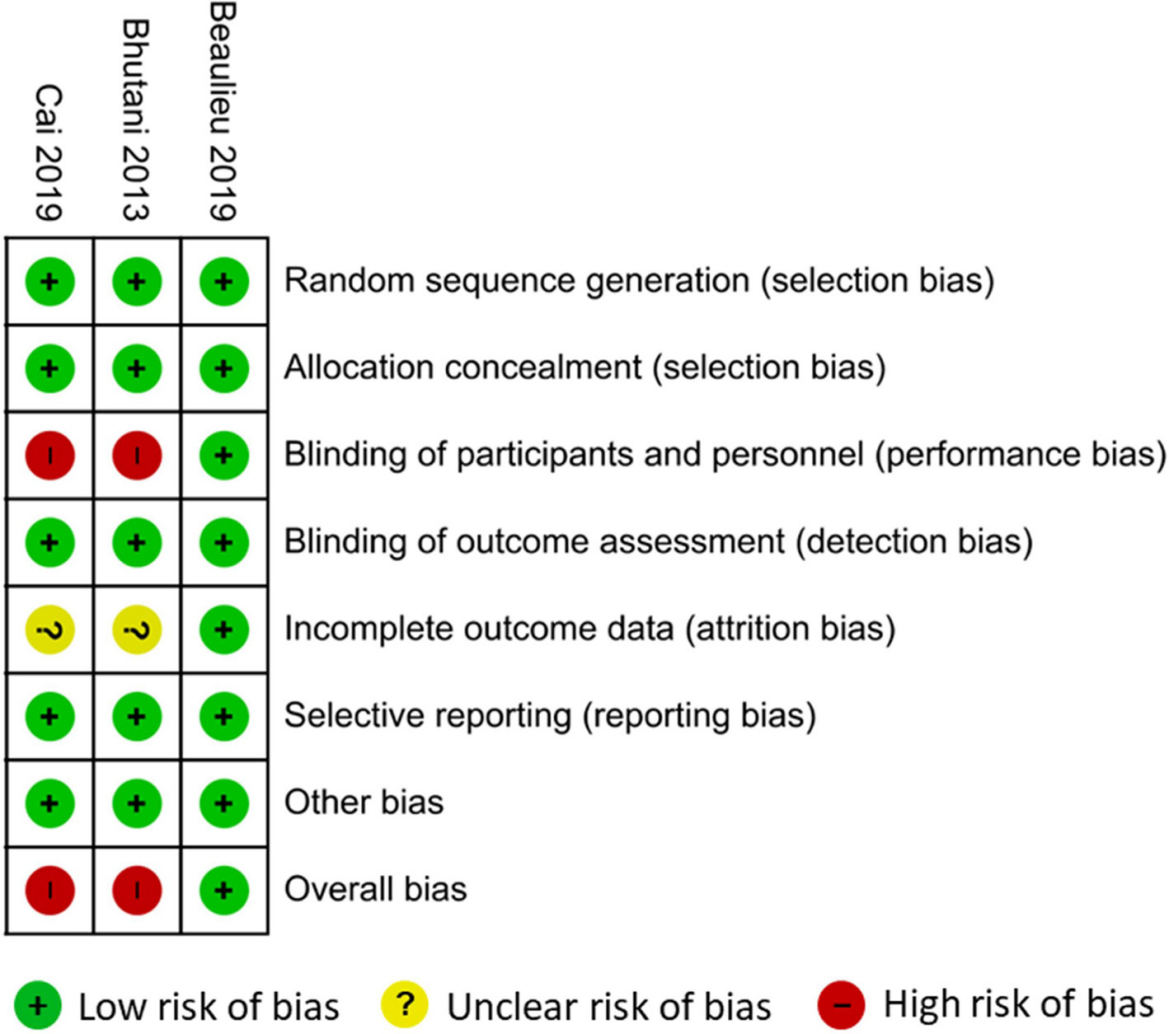
Risk of bias assessments of the included RCTs.

**Fig. 3.**
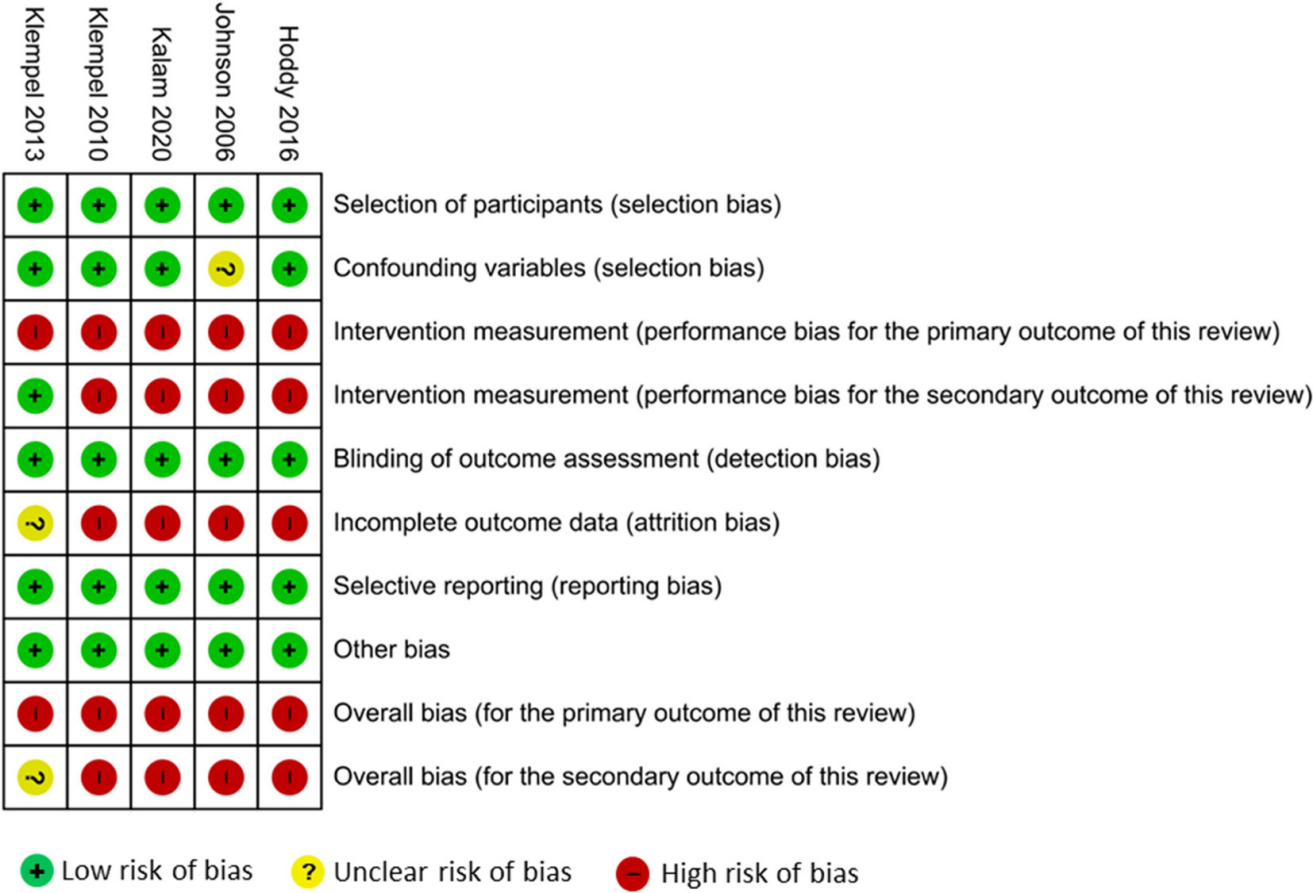
Risk of bias assessments of the included UCBA studies.

The risk of bias assessments are summarised in Figs. 2 and 3. With respect to the three RCTs, two RCTs gave no information about blinding of participants and providers^(31,48)^. For the same two studies, there was also some concern with respect to high loss to follow-up (attrition bias). Thus, overall, we judged that these two RCTs^(31,48)^ had high risk of bias while the study by Beaulieu *et al.*^(16)^ had low risk of bias. In that study, the outcome assessors remained blinded to the diet allocations throughout the entire intervention and the participants were not aware of the true aims of the study.^(16)^ For the five UCBA studies, there was some risk of bias with respect to performance bias and incomplete outcome data. Thus, for the primary outcome, we judged that all had high risk of bias, and all except Klempel *et al.*^(47)^ had high risk of bias also with respect to the secondary outcome.

### Effects of ADF on appetite

**Fig. 4.**
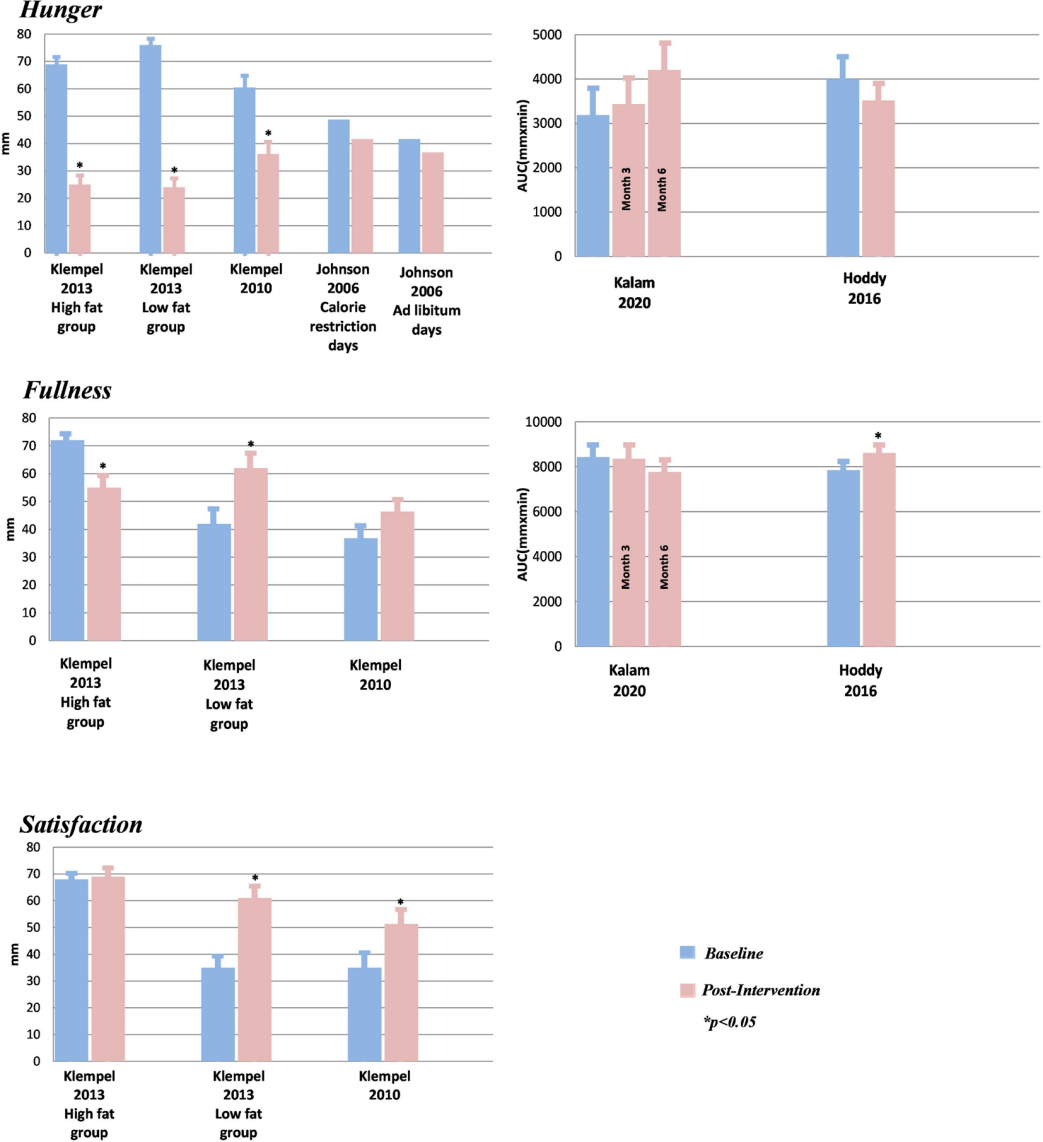
Feelings of appetite from baseline to follow-up in the UCBA studies. UCBA, uncontrolled before-after study; AUC, area under curve; mm, millimetre; min, minute. *Statistically significant difference from baseline to post-intervention (*P* < 0·05). Values are reported as mean and standard error of the mean.

With respect to hunger, none of the RCTs detected a significant difference between ADF and the control group at the 12 weeks follow-up (Table 3). While three of the UCBA studies found no significant difference from baseline to follow-up, in the two studies by Klempel *et al.*^(47,51)^, hunger decreased significantly from baseline to follow-up (Table 4, Fig. 4).

**Table 3.** Between-group differences for the outcomes in RCTs

	ADF group						Control group						Result/Effect Estimate between groups	
Outcome/Study	Baseline (mean)	sem	Post-intervention (mean)	sem	Mean change	sem	Baseline (mean)	sem	Post-intervention (mean)	sem	Mean change	sem		Between-group difference *P* value
Hunger (VAS 0–100 mm)														
Beaulieu 2019	NR	NR	NR	NR	NR	NR	NR	NR	NR	NR	NR	NR	NS	*P* ≥ 0·05
Bhutani 2013	6·3	0·5	4·7	0·7	−1·6	0·7	NR	NR	NR	NR	0	0	NS	*P* ≥ 0·05
Cai 2019*	30·0	0·4	14·0	0·2	−16·0	0·1	25·0	0·3	11·0	0·2	−14·0	0·1	NS	*P* ≥ 0·05
Fullness (VAS 0–100 mm)														
Beaulieu 2019	NR	NR	NR	NR	NR	NR	NR	NR	NR	NR	NR	NR	NS	*P* ≥ 0·05
Bhutani 2013	2·4	0·4	4·0	0·7	1·6	0·6	NR	NR	NR	NR	0	0	NS	*P* ≥ 0·05
Cai 2019*	4·0	0·1	19·0	0·1	15·0	0·1	8·0	0·1	16·0	0·1	8·0	0·1	NS	*P* ≥ 0·05
Satisfaction (VAS 0–100 mm)														
Bhutani 2013	3·2	0·4	4·3	0·6	1·1	0·5	NR	NR	NR	NR	0	0	NS	*P* ≥ 0·05
Cai 2019*	27·0	0·6	35·0	0·4	8·0	0·2	23·0	0·5	28·0	0·6	4·0	0·1	NS	*P* ≥ 0·05
Desire to eat (VAS 0–100 mm)														
Beaulieu 2019	NR	NR	NR	NR	NR	NR	NR	NR	NR	NR	NR	NR	NS	*P* ≥ 0·05
Body weight (kg)														
Beaulieu 2019	81·2	2·7	NR	NR	NR	NR	78·6	2·1	NR	NR	NR	NR	NS	*P* ≥ 0·05
Bhutani 2013	94·0	3·0	NR	NR	−3·0	1·0	93·0	5	NR	NR	−1·9	0·07	NS	*P* ≥ 0·05
Cai 2019	75·3	0·9	71·3	0·7	−4·0	0·06	72·9	0·9	71·8	0·8	−1·9	0·07	NS	*P* ≥ 0·05

RCT, randomised controlled trial; ADF, alternate day fasting; sem, standard error of the mean; VAS, visual analogue scale; mm, millimetre; NR, not reported; NS, no significant difference; kg, kilogram.

* Baseline values were not reported and week 4 values were used in the table.

**Table 4.** Within-group differences for the outcomes in UCBA studies

Outcome/Study	Baseline (mean)	sem	Post-intervention (ADF) (mean)	sem	Result/Effect Estimate from baseline to follow-up	Difference within group *P* value
Hunger (VAS 0–100 mm)						
Klempel 2013	HF group: 69·0	HF group: 3·0	HF group: 25·0	HF group: 4·0	Sign. diff. from baseline to follow-up	*P* < 0·05
	LF group: 76·0	LF group: 3·0	LF group: 24·0	LF group: 4·0	Sign. diff. from baseline to follow-up	*P* < 0·05
Klempel 2010	60·5	5·0	36·2	5·0	Sign. diff. from baseline to follow-up	*P* < 0·05
Hunger (Numeric rating scale 1–10)						
Johnson 2006	CR day: 5·4	CR day: 6·0	CR day: 4·8	CR day: 5·4	NS	*P* > 0·05
	AL day: 4·8	AL day: 5·2	AL day: 4·3	AL day: 4·9	NS	*P* > 0·05
Hunger (AUC mm × min)						
Kalam 2020	3187·8	660·6	Month 3: 3437·4	Month 3: 644·6	NS	*P* > 0·05
			Month 6: 4209·1	Month 6: 669·5	NS	*P* > 0·05
Hoddy 2016	4000·2	464·0	3518·2	418·6	NS	*P* > 0·05
Fullness (VAS 0–100 mm)						
Klempel 2013	HF group: 72·0	HF group: 3·0	HF group: 55·0	HF group: 5·0	Sign. diff. from baseline to follow-up	*P* < 0·05
	LF group: 42·0	LF group: 6·0	LF group: 62·0	LF group: 6·0	Sign. diff. from baseline to follow-up	*P* < 0·05
Klempel 2010	36·8	5·1	46·4	5·0	NS	*P* > 0·05
Fullness (AUC mm × min)						
Kalam 2020	8435·6	651·5	Month 3: 8357·6	Month 3: 679·6	NS	*P* > 0·05
			Month 6: 7763·7	Month 6: 666·9	NS	*P* > 0·05
Hoddy 2016	7849·4	482·4	8610·8	462·0	Sign. Diff. from baseline to follow-up	*P* < 0·05
Satisfaction (VAS 0–100 mm)						
Klempel 2013	HF group: 68·0	HF group: 3·0	HF group: 69·0	HF group: 4·0	NS	*P* > 0·05
	LF group: 35·0	LF group: 5·0	LF group: 61·0	LF group: 5·0	Sign. diff. from baseline to follow-up	*P* < 0·05
Klempel 2010	35·0	6·0	51·3	6·0	Sign. Diff. from baseline to follow-up	*P* < 0·05
Body weight change (kg)						
	Mean Change (baseline to post-intervention		sem			
Kalam 2020*	Month 3: −5·3		Month 3: 0·6		Sign. diff. from baseline to follow-up	*P* < 0·001
	Month 6: −6·2		Month 6: 1·0		Sign. diff. from baseline to follow-up	*P* < 0·001
Hoddy 2016	−3·9		0·6		Sign. diff. from baseline to follow-up	*P* < 0·0001
Klempel 2013	HF group: 4·4		HF group: 1·0		Sign. diff. from baseline to follow-up	*P* < 0·0001
	LF group: 3·7		LF group: 0·7		Sign. diff. from baseline to follow-up	*P* < 0·0001
Klempel 2010	−5·6		1·0		Sign. diff. from baseline to follow-up	*P* < 0·001
Johnson 2006	−8·5		1·7^†^		Sign. diff. from baseline to follow-up	*P* < 0·01

UCBA, uncontrolled before-after study; sem, standard error of the mean; ADF, alternate day fasting; VAS, visual analogue scale; mm, millimetre; HF, high-fat; sign., significant; diff., difference; LF, low-fat; CR, caloric-restriction; NS, no significant difference; AL, *ad libitum*; AUC, area under curve; min, minute; kg, kilogram.

* There was no statistically significant difference between month 3 and month 6.

† It was not stated in the study whether it is standard deviation or sem.

Concerning fullness, none of the RCTs found a significant difference between the groups. In the study by Beaulieu *et al.*^(16)^, although they found no significant difference between groups in their intention-to-treat analysis, as shown in Table 3, in their completers analysis, there was an increase in fullness AUC after the intervention (*M*_Δ_ = 781 (95 % CI −0·2, 1563) mm × min, *P* = 0·05) and a difference between groups, with ADF having greater fullness overall than CER (*M*_Δ_ = 1555 (95 % CI 46, 3064) mm × min, *P* = 0·04). Of the UCBA studies, two found no significant changes while the other two found somewhat contradictory changes (Table 4, Fig. 4). While Hoddy *et al.*^(22)^ detected a significant increase from baseline to follow-up, Klempel *et al.*^(47)^ detected a significant increase in the low-fat diet group but a significant decrease in the high-fat diet group.

Four studies reported on satisfaction. Neither of the two RCTs found a significant difference between the groups at follow-up. Both studies by Klempel *et al.*^(47,51)^ found a significant increase in satisfaction from baseline to follow-up, except for in the low-fat diet group, which had no significant change during the intervention period (Table 4, Fig. 4).

Only one study reported on desire to eat. The RCT by Beaulieu *et al.*^(16)^ found no significant difference between the ADF group and the CER group (Table 3).

### Effects of ADF on body weight

As seen in Table 3, for body weight, none of the RCTs found a significant difference between the ADF group and the control group at 12 weeks follow-up. The five UCBA studies all detected a significant decrease in body weight from baseline to follow-up (Table 4). Overall, across 8–10 weeks, body weight decreased from 3·7 kg to 8·5 kg (mode 4·4 kg) (Table 4). Kalam *et al.*^(50)^ had the longest follow-up and detected similar decreases at 3 months follow-up (5·3 kg) and at 6 months follow-up (6·2 kg) (Table 4).

### Certainty of the evidence

The overall certainty of the evidence was very low for all outcomes except desire to eat which was rated as low certainty (Table 5).

**Table 5.** Summary of findings (GRADE)

**Population**: Patients with overweight or obesity (BMI ≥ 25 kg/m^2^) **Intervention**: Alternate day fasting (ADF) **Comparison**: Continuous energy restriction and no specified diet **Countries**: China, England, USA			
**Outcome, follow-up time**	**Effect**	**No. of participants (Studies)**	**Quality of evidence (GRADE)**
Appetite (8–24 weeks)			
Hunger	Six of eight studies (three RCTs, three UCBA studies) found no sign. diff.; two UCBA studies found sign. decrease (Tables 3 and 4, Fig. 4)	456 (8)	⨁◯◯◯ VERY LOW^a^
Fullness	Five of seven studies (three RCTs, two UCBA studies) found no sign. diff.; two UCBA studies found sign. decrease and increase (Tables 3 and 4, Fig. 4)	442 (7)	⨁◯◯◯ VERY LOW^a^
Satisfaction	Two RCTs found no sign. diff. and two UCBA studies found sign. increase (Tables 3 and 4, Fig. 4)	270 (4)	⨁◯◯◯ VERY LOW^a^
Desire to eat	One RCT found no sign. diff. (Table 3)	46 (1)	⨁⨁◯◯ LOW^b^
Body weight (8–24 weeks)			
Three RCTs found no sign. diff. and five UCBA studies found sign. decrease (Tables 3 and 4)		456 (8)	⨁◯◯◯ VERY LOW^c^

GRADE, grading of recommendations assessment, development and evaluation; BMI, body mass index; ADF, alternate day fasting; RCT, randomised controlled trial; UCBA, uncontrolled before-after study; sign., significant; diff., difference.

a Downgraded by three levels because of risk of bias, imprecision and inconsistency.

b Downgraded by two levels because of imprecision.

c Downgraded by three levels because of risk of bias and imprecision.

## Discussion

This systematic review advances the evidence on the effects of ADF for overweight and obese patients. To the best of our knowledge, this is the first systematic review with a focus on the effects of ADF on subjective feelings of appetite for overweight and obese adults. There are numerous reviews that summarise the effects of IER approaches and also just ADF on body weight, our secondary outcome^(20,24,25,28–30,52–56)^. Our findings are in line with these previous reviews and document that there is no conclusive evidence, but that positive short-term effects of ADF are indicated for body weight.

The included RCTs found neither significant unfavourable effects, such as increases in the feelings of hunger, nor significant benefits of ADF over CER for any of the outcomes after 3 months. Meanwhile, some of the UCBA studies detected some significant improvements during the intervention period, most notably in body weight. Mirroring results in two related systematic reviews^(28,55)^, with respect to body weight, all the included studies in our systematic review showed reductions in the range of 3–9 kilos during the intervention period. For dietary interventions, The American Dietetic Associations' Adult Weight Maintenance Evidence Based Nutrition Practice Guideline recommends an optimal weight loss target of 0·5–1 kg per week for the first 6 months^(57)^, which is in line with the findings of our review. Typically, diet-induced weight loss in overweight and obese adults is accompanied by an increase in hunger and desire to eat, and a decrease in fullness and satisfaction^(11,58,59)^. Our results suggest that it is uncertain whether ADF has an effect on the typical subjective feelings of appetite associated with dietary prescriptions, and further research is needed to understand the effects of ADF better.

### Overall completeness and applicability of the evidence

The certainty of the evidence for all outcomes in this systematic review is generally very low, and the results must be interpreted cautiously. This is mainly because five of the eight studies had no control group, most studies had considerable risk of bias and there was imprecision in effects. Across the eight studies, sample sizes were small, and in total, there were only 456 study participants. Some of the included studies may have been inadequately powered to detect differences as only two studies reported power calculations^(16,50)^. Only one included study followed participants beyond 3 months and results beyond this time are uncertain. We note, however, that Kalam *et al.*^(50)^ had 6 months follow-up, with results being similar at 3 and 6 months. Furthermore, we included only studies on adults with overweight or obesity of BMI ≥25 kg/m^2^, receiving ADF consisting of a cyclical feeding pattern with a fast day (0–25 % of the individuals' daily energy needs) alternated with a feast day. Yet, there were considerable heterogeneity in sample and intervention characteristics, including various ADF components such as prescribed energy intake, macronutrient composition, timing of consumption of foods during fast days and provision of additional dietary counselling. The transferability of the findings is therefore uncertain. Consistent with similar reviews^(55,60)^, participants were predominantly female, which supports the assertion that there is a gender imbalance with respect to weight management programme engagement.

### Implications

The very low certainty of evidence on the effects of ADF for overweight and obese patients prevents strong recommendations. Therefore, ADF should be offered cautiously in clinical practice while concomitantly evaluating outcomes important for patients. Given the complexity of weight management, it is unlikely that a ‘one size fits all’ approach will work.

Prior to recommending ADF to overweight and obese patients, more trials are needed to be certain of its benefits. Patient groups should be involved in ADF implementation and evaluation, to maximise the potential for modification and ultimately effect. For example, examining the adherence to ADF in relation to cognitive, environmental, psychological, and physiological factors and identifying barriers and facilitators will help to better comprehend the effects of ADF and assess feasibility. Similarly, evaluations of the impact of ADF on appetite hormones such as ghrelin, PYY or glucagon-like peptide 1 (GLP-1), and ketone bodies during weight loss and weight maintenance are needed. Our systematic review suggests that this is an important area for further research. Although all the included studies assessed ADF consisting of a cyclical feeding pattern with a fast day alternated with a feast day, the macronutrient compositions of the ADF intervention differed across the studies. In order to allow for comparisons among studies and to translate the research evidence into practice, there is a need to standardise the key elements of ADF, as well as to harmonise outcomes and improve reporting of studies examining these outcomes. Future trials of ADF should average measurements taken from consecutive feed and fast days throughout the day to attain accurate assessments. This systematic review advocates for methodologically rigorous, adequately powered, long-term, large-scale RCTs conducted in different settings with diverse populations in preferably free-living conditions.

### Strengths and limitations

Our systematic review was conducted in accordance with international guidelines for systematic reviews, the researchers are experienced in conducting systematic reviews, and one researcher is a registered dietician with long and diverse experience. As encouraged by others, due to the limited number of ADF studies published to date^(55,61)^, we included not only RCTs, thus preventing a potential loss of critical information. However, we excluded conference abstracts, studies with no accessible full-texts, and studies in languages other than English, and it is possible that relevant studies have been missed and or have been published after our search. Due to clinical and methodological heterogeneity and missing and differentially reported outcome data, we could not conduct meta-analyses.

## Conclusion

This systematic review found low to very low evidence on the effects of ADF, in comparison to CER and *ad libitum* diet, on subjective feelings of hunger and body weight for adult patients with overweight and obesity. Additional trials are needed to be certain of the benefits of ADF, and if offered in clinical practice, it should be offered cautiously while concomitantly evaluated. Despite the high interest in ADF, good quality evidence is still needed to determine its effectiveness.

## Supplementary material

For supplementary material accompanying this paper visit https://doi.org/10.1017/jns.2022.90. click here to view supplementary material
