# Supplementary material for: Alternate day fasting on subjective feelings of appetite and body weight for adults with overweight or obesity: a systematic review
Source: J Nutr Sci. 2022 Oct 31;11:e94. doi: 10.1017/jns.2022.90 (PMC9641525; doi:10.1017/jns.2022.90)
Supplement: Supplementary file 1 [file S2048679022000908sup001.docx]

**Appendices/Supplementary materials**

**Appendix 1. Full search strategy**

Embase Classic+Embase (1947 to February 20, 2021) - 4319 hits

1. obes*.ti,ab,kw.

2. exp *obesity/

3. overweight*.ti,ab,kw.

4. *overnutrition/

5. overnutrition.ti,ab,kw.

6. fatness.ti,ab,kw.

7. body weight.ti.

8. exp *patient/

9. exp *adult/

10. patient*.ti,ab,kw.

11. adult*.ti,ab,kw.

12. female*.ti,ab,kw.

13. male*.ti,ab,kw.

14. wom#n.ti,ab,kw.

15. m#n.ti,ab,kw.

16. *fasting/

17. *intermittent fasting/

18. *alternate day fasting/

19. (fasting not ((fasting adj2 blood) or (fasting adj2 glucose) or (fasting adj2 insulin))).ti,ab,kw.

20. fasting therap*.ti,ab,kw.

21. intermittent fast*.ti,ab,kw.

22. alternate day fast*.ti,ab,kw.

23. periodic fast*.ti,ab,kw.

24. (alternate day adj1 modified fast*).ti,ab,kw.

25. intermittent energy restrict*.ti,ab,kw.

26. intermittent calori# restrict*.ti,ab,kw.

27. alternate day calori# restrict*.ti,ab,kw.

28. every other day fast*.ti,ab,kw.

29. *body weight/

30. *body size/

31. *body fat/

32. *body mass/

33. *body composition/

34. exp *body weight change/

35. *body weight control/

36. exp *body weight management/

37. body siz*.ti,ab,kw.

38. body composition.ti,ab,kw.

39. fat mass.ti,ab,kw.

40. body fat.ti,ab,kw.

41. weight.ti,ab,kw.

42. fat loss*.ti,ab,kw.

43. body mass.ti,ab,kw.

44. *appetite/

45. *increased appetite/

46. *decreased appetite/

47. *"loss of appetite"/

48. exp *food craving/

49. craving*.ti,ab,kw.

50. *hunger/

51. *satiety/

52. *satiety response/

53. appetite.ti,ab,kw.

54. feeling*.ti,ab,kw.

55. hunger.ti,ab,kw.

56. fullness.ti,ab,kw.

57. hungry.ti,ab,kw.

58. satiety.ti,ab,kw.

59. satiation.ti,ab,kw.

60. desire to eat.ti,ab,kw.

61. eating attitude*.ti,ab,kw.

62. eating behavio?r*.ti,ab,kw.

63. (news or editorial or comment).pt.

64. 1 or 2 or 3 or 4 or 5 or 6 or 7

65. 8 or 9 or 10 or 11 or 12 or 13 or 14 or 15

66. 64 and 65

67. 16 or 17 or 18 or 19 or 20 or 21 or 22 or 23 or 24 or 25 or 26 or 27 or 28

68. 29 or 30 or 31 or 32 or 33 or 34 or 35 or 36 or 37 or 38 or 39 or 40 or 41 or 42 or 43 or 44 or 45 or 46 or 47 or 48 or 49 or 50 or 51 or 52 or 53 or 54 or 55 or 56 or 57 or 58 or 59 or 60 or 61 or 62

69. 66 and 67 and 68

70. 69 not 63

71. limit 70 to yr="2000-2020"

72. limit 71 to human

73. limit 72 to english language

Last Searched: 20.02.2021

MEDLINE In‐Process & Other Non‐Indexed Citations Ovid (1946 to February 20, 2021) – 2249 hits

1. obes*.ti,ab,kw.

2. exp *overweight/

3. *overnutrition/

4. overweight*.ti,ab,kw.

5. overnutrition.ti,ab,kw.

6. fatness.ti,ab,kw.

7. body weight.ti.

8. exp *patients/

9. exp *adult/

10. patient*.ti,ab,kw.

11. adult*.ti,ab,kw.

12. female*.ti,ab,kw.

13. male*.ti,ab,kw.

14. wom#n.ti,ab,kw.

15. m#n.ti,ab,kw.

16. *fasting/

17. (fasting not ((fasting adj2 blood) or (fasting adj2 glucose) or (fasting adj2 insulin))).ti,ab,kw.

18. fasting therap*.ti,ab,kw.

19. intermittent fast*.ti,ab,kw.

20. alternate day fast*.ti,ab,kw.

21. periodic fast*.ti,ab,kw.

22. (alternate-day adj1 modified fast*).ti,ab,kw.

23. intermittent energy restrict*.ti,ab,kw.

24. intermittent calori# restrict*.ti,ab,kw.

25. alternate day calori# restrict*.ti,ab,kw.

26. every other day fast*.ti,ab,kw.

27. *body size/

28. *body weight/

29. *body composition/

30. *body mass index/

31. exp *body weight changes/

32. body siz*.ti,ab,kw.

33. body composition.ti,ab,kw.

34. fat mass.ti,ab,kw.

35. body fat.ti,ab,kw.

36. weight.ti,ab,kw.

37. fat loss*.ti,ab,kw.

38. body mass.ti,ab,kw.

39. *appetite/

40. *appetite regulation/

41. *craving/

42. *hunger/

43. exp *satiation/

44. appetite.ti,ab,kw.

45. feeling*.ti,ab,kw.

46. craving*.ti,ab,kw.

47. hunger.ti,ab,kw.

48. fullness.ti,ab,kw.

49. hungry.ti,ab,kw.

50. satiety.ti,ab,kw.

51. satiation.ti,ab,kw.

52. desire to eat.ti,ab,kw.

53. eating attitude*.ti,ab,kw.

54. eating behavio?r*.ti,ab,kw.

55. (news or editorial or comment).pt.

56. 1 or 2 or 3 or 4 or 5 or 6 or 7

57. 8 or 9 or 10 or 11 or 12 or 13 or 14 or 15

58. 56 and 57

59. 16 or 17 or 18 or 19 or 20 or 21 or 22 or 23 or 24 or 25 or 26

60. 27 or 28 or 29 or 30 or 31 or 32 or 33 or 34 or 35 or 36 or 37 or 38 or 39 or 40 or 41 or 42 or 43 or 44 or 45 or 46 or 47 or 48 or 49 or 50 or 51 or 52 or 53 or 54

61. 58 and 59 and 60

62. 61 not 55

63. limit 62 to yr="2000-2020"

64. limit 63 to human

65. limit 64 to english language

Last Searched: 20.02.2021

APA PsycINFO (1806 to February 20, 2021) – 200 hits

1. obes*.ti,ab,id.

2. exp *"Obesity (Attitudes Toward)"/

3. overnutrition.ti,ab,id.

4. exp *overweight/

5. overweight*.ti,ab,id.

6. fatness.ti,ab,id.

7. body weight.ti.

8. exp *patients/

9. patient*.ti,ab,id.

10. adult*.ti,ab,id.

11. female*.ti,ab,id.

12. male*.ti,ab,id.

13. wom#n.ti,ab,id.

14. m#n.ti,ab,id.

15. (fasting not ((fasting adj2 blood) or (fasting adj2 glucose) or (fasting adj2 insulin))).ti,ab,id.

16. fasting therap*.ti,ab,id.

17. intermittent fast*.ti,ab,id.

18. alternate day fast*.ti,ab,id.

19. (alternate-day adj1 modified fast*).ti,ab,id.

20. periodic fast*.ti,ab,id.

21. intermittent energy restrict*.ti,ab,id.

22. intermittent calori# restrict*.ti,ab,id.

23. alternate day calori# restrict*.ti,ab,id.

24. every other day fast*.ti,ab,id.

25. *body fat/

26. *body size/

27. *body weight/

28. *body mass index/

29. *weight control/

30. *weight gain/

31. *weight loss/

32. body siz*.ti,ab,id.

33. body composition.ti,ab,id.

34. fat mass.ti,ab,id.

35. body fat.ti,ab,id.

36. weight.ti,ab,id.

37. fat loss*.ti,ab,id.

38. body mass.ti,ab,id.

39. exp *appetite/

40. *craving/

41. *eating attitudes/

42. appetite.ti,ab,id.

43. craving*.ti,ab,id.

44. feeling*.ti,ab,id.

45. hunger.ti,ab,id.

46. fullness.ti,ab,id.

47. hungry.ti,ab,id.

48. satiety.ti,ab,id.

49. satiation.ti,ab,id.

50. desire to eat.ti,ab,id.

51. eating attitude*.ti,ab,id.

52. eating behavio?r*.ti,ab,id.

53. (news or editorial or comment).pt.

54. 1 or 2 or 3 or 4 or 5 or 6 or 7

55. 8 or 9 or 10 or 11 or 12 or 13 or 14

56. 54 and 55

57. 15 or 16 or 17 or 18 or 19 or 20 or 21 or 22 or 23 or 24

58. 25 or 26 or 27 or 28 or 29 or 30 or 31 or 32 or 33 or 34 or 35 or 36 or 37 or 38 or 39 or 40 or 41 or 42 or 43 or 44 or 45 or 46 or 47 or 48 or 49 or 50 or 51 or 52

59. 56 and 57 and 58

60. 59 not 53

61. limit 60 to yr="2000-2020"

62. limit 61 to human

63. limit 62 to english language

Last Searched: 20.02.2021

EBSCOhost CINAHL – 76 hits

S1. (MM “obesity+”)

S2. TI obes* OR AB obes*

S3. TI overweight* OR AB overweight*

S4. TI overnutrition OR AB overnutrition

S5. TI fatness OR AB fatness

S6. TI “body weight”

S7. (MM “patients+”)

S8. (MM “adult+”)

S9. TI patient* OR AB patient*

S10. TI adult* OR AB adult*

S11. TI male* OR AB male*

S12. TI female* OR AB female*

S13. TI wom?n OR AB wom?n

S14. TI m?n OR AB m?n

S15. (MM “fasting”)

S16. TI “fasting therap*” OR AB “fasting therap*”

S17. TI fasting OR AB fasting

S18. TI (fasting) W2 (blood) OR AB (fasting) W2 (blood)

S19. TI (fasting) W2 (glucose) OR AB (fasting) W2 (glucose)

S20. TI (fasting) W2 (insulin) OR AB (fasting) W2 (insulin)

S21. S18 OR S19 OR S20

S22. S17 NOT S21

S23. TI “intermittent fast*” OR AB “intermittent fast*”

S24. TI “alternate day fast*” OR AB “alternate day fast*”

S25. TI “alternate day calori? restrict*” OR AB “alternate day calori? restrict*”

S26. TI “periodic fast*” OR AB “periodic fast*”

S27. TI (modified N1 alternate-day fast*) OR AB (modified N1 alternate-day fast*)

S28. TI “intermittent energy restrict*” OR AB “intermittent energy restrict*”

S29. TI “intermittent calori? restrict*” OR AB “intermittent calori? restrict*”

S30. TI “every other day fast*” OR AB “every other day fast*”

S31. (MM “body size”)

S32. (MM “body weight”)

S33. (MM “body weight changes”)

S34. (MM”body composition”)

S35. (MM “body mass index”)

S36. (MM “weight control”)

S37. (MM “weight gain”)

S38. (MM “weight loss”)

S39. TI “body composition” OR AB “body composition”

S40. TI “body siz*” OR AB “body siz*”

S41. TI “fat mass” OR AB “fat mass”

S42. TI “body fat” OR AB “body fat”

S43. TI weight OR AB weight

S44. TI “fat loss*” OR AB “fat loss*”

S45. TI “body mass” OR AB “body mass”

S46. (MM “appetite”)

S47. (MM “hunger”)

S48. (MM “satiation”)

S49. (MM “craving”)

S50. TI hunger OR AB hunger

S51. TI appetite OR AB appetite

S52. TI fullness OR AB fullness

S53. TI hungry OR AB hungry

S54. TI satiety OR AB satiety

S55. TI satiation OR AB satiation

S56. TI “desire to eat” OR AB “desire to eat”

S57. TI craving* OR AB craving*

S58. TI feeling* OR AB feeling*

S59. TI “eating attitude*” OR AB “eating attitude*”

S60. TI eating behavio#r* OR AB eating behavio#ur*

S61. S1 OR S2 OR S3 OR S4 OR S5 OR S6

S62. S7 OR S8 OR S9 OR S10 OR S11 OR S12 OR S13 OR S14

S63. S61 AND S62

S64. S15 OR S16 OR S22 OR S23 OR S24 OR S25 OR S26 OR S27 OR S28 OR S29 OR S30

S65. S31 OR S32 OR S33 OR S34 OR S35 OR S36 OR S37 OR S38 OR S39 OR S40 OR S41 OR S42 OR S43 OR S44 OR S45 OR S46 OR S47 OR S48 OR S49 OR S50 OR S51 OR S52 OR S53 OR S54 OR S55 OR S56 OR S57 OR S58 OR S59 OR S60

S66. S63 AND S64 AND S65

Limiters : Date of publication (2000/01/01 – 2020/12/31), Human, English language

Last Searched: 20.02.2021

Google Scholar – 136 hits

allintitle: "alternate day fasting"

Year: 2000 to 2020

Last Searched: 20.02.2021

**Appendix 2. Studies excluded in full-text assessments with reasons**

| **Study** | | **Reason for exclusion** |
| --- | --- | --- |
|  | Akasheh RT, Kroeger CM, Trepanowski JF, Gabel K, Hoddy KK, Kalam F, et al. Weight loss efficacy of alternate day fasting versus daily calorie restriction in subjects with subclinical hypothyroidism: a secondary analysis. Applied Physiology, Nutrition, & Metabolism = Physiologie Appliquee, Nutrition et Metabolisme. 2020;45(3):340-3. | No data for the primary outcome of this review |
|  | Ash S, Reeves MM, Yeo S, Morrison G, Carey D, Capra S. Effect of intensive dietetic interventions on weight and glycaemic control in overweight men with Type II diabetes: a randomized trial. International Journal of Obesity & Related Metabolic Disorders: Journal of the International Association for the Study of Obesity. 2003;27(7):797-802. | Type of intervention – IER for 4 consecutive days per week |
|  | Beaulieu K, Casanova N, Oustric P, Gibbons C, Hopkins M, Varady K, et al. Matched Weight Loss through Intermittent or Continuous Energy Restriction Does Not Result in Compensatory Adaptations in Appetite: A Proof of Concept Rct. Clinical Nutrition. 2019;38:S22. | Different publication of one of the included article (Beaulieu et al. 2019) |
|  | Beaulieu K, Casanova N, Oustric P, Gibbons C, Hopkins M, Varady K, et al. Proof of concept RCT investigating the impact of matched weight loss via intermittent or continuous energy restriction on appetite control. Obesity Facts. 2019;12:12. | Different publication of one of the included article (Beaulieu et al. 2019) |
|  | Beaulieu K, Casanova N, Oustric P, Hopkins M, Varady K, Finlayson G, et al. An exploratory investigation of the impact of "fast" and "feed" days during intermittent energy restriction on free-living energy balance behaviours and subjective states in women with overweight/obesity. European Journal of Clinical Nutrition. 2020. | Different publication of one of the included article (Beaulieu et al. 2019) |
|  | Bhutani S, Klempel MC, Berger RA, Varady KA. Improvements in coronary heart disease risk indicators by alternate-day fasting involve adipose tissue modulations. Obesity. 2010;18(11):2152-9. | No data for the primary outcome of this review |
|  | Bhutani S, Klempel MC, Kroeger CM, Trepanowski JF, Varady KA. Alternate day fasting and endurance exercise combine to reduce body weight and favorably alter plasma lipids in obese humans. Obesity. 2013;21(7):1370-9. | No data for the primary outcome of this review  - Secondary analysis of this study is included in the review (Bhutani et al. 2013) |
|  | Bhutani S, Klempel MC, Kroeger CM, Varady KA. OP024 COMBINING ALTERNATE DAY FASTING WITH EXERCISE IMPROVES PLASMA LIPIDS AND LDL PARTICLE SIZE IN OBESE HUMANS. Clinical Nutrition Supplements. 2012;1(7):10-1. | Conference abstract |
|  | Casanova N, Beaulieu K, Oustric P, Gibbons C, Finlayson G, Hopkins M. Reductions in Physical Activity Attenuate the Rate of Weight Loss during Dietary Energy Restriction in Women with Overweight and Obesity. Clinical Nutrition. 2019;38:S308. | Conference abstract |
|  | Catenacci VA, Pan Z, Ostendorf D, Brannon S, Gozansky WS, Mattson MP, et al. A randomized pilot study comparing zero-calorie alternate-day fasting to daily caloric restriction in adults with obesity. Obesity (19307381). 2016;24(9):1874-83. | No data for the primary outcome of this review |
|  | Cho AR, Moon JY, Kim S, An KY, Oh M, Jeon JY, et al. Effects of alternate day fasting and exercise on cholesterol metabolism in overweight or obese adults: A pilot randomized controlled trial. Metabolism: Clinical and Experimental. 2019;93:52-60. | No data for the primary outcome of this review |
|  | Chun JY, Seo YH. The Effects of a 10-week Alternate Day Fasting (ADF) and Combined Exercise Program on Body Composition, Physical Fitness and Blood Variables in Middle-aged Women. 한국체육학회 기타간행물. 2013:342-. | Full-text not found |
|  | E OS. White Blood Cell and Hemoglobin Response in Combination Alternate Day Fasting and Exercise Interventions: University of Illinois at Chicago; 2010. | Full-text not found |
|  | Gabel K, Kroeger CM, Trepanowski JF, Hoddy KK, Cienfuegos S, Kalam F, et al. Differential Effects of Alternate-Day Fasting Versus Daily Calorie Restriction on Insulin Resistance. Obesity. 2019;27(9):1443-50. | No data for the primary outcome of this review |
|  | Gumpricht E, Ward E, He F, Schuler B, Cho S, Arciero P. Effects of a high-protein low-calorie intermittent-fast diet on plasma toxins and oxidative stress following weight loss. FASEB Journal Conference: Experimental Biology. 2014;28(1). | Conference Abstract |
|  | Gunasekaran D, Dinary BA. The effects of intermittent fasting on the progression of CKD. Journal of the American Society of Nephrology. 2020;31:189-90. | Conference Abstract |
|  | He F, Zuo L, Emery W, Arciero P. High protein intermittent fasting increases serum polychlorinated biphenyls and decreases oxidative stress in obese adults. Journal of Hypertension. 2017;35:e258. | Conference Abstract |
|  | Hoddy K, Kroeger C, Trepanowski J, Bhutani S, Barnosky A, Varady K. Meal timing during alternate day fasting: Effects on body weight and coronary heart disease risk in obese adults. FASEB Journal Conference: Experimental Biology. 2014;28(1). | No data for the primary outcome of this review  -Secondary analysis of this study is included in the review (Hoddy et al. 2016) |
|  | Hoddy KK, Bhutani S, Phillips SA, Varady KA. Effects of different degrees of insulin resistance on endothelial function in obese adults undergoing alternate day fasting. Nutrition and healthy aging. 2016;4(1):63-71. | No data for the primary outcome of this review |
|  | Hoddy KK, Kroeger CM, Trepanowski JF, Barnosky A, Bhutani S, Varady KA. Meal timing during alternate day fasting: Impact on body weight and cardiovascular disease risk in obese adults. Obesity. 2014;22(12):2524-31. | No data for the primary outcome of this review |
|  | Hoddy KK, Kroeger CM, Trepanowski JF, Barnosky AR, Bhutani S, Varady KA. Safety of alternate day fasting and effect on disordered eating behaviors. Nutrition Journal. 2015;14(1). | No data for the primary outcome of this review |
|  | Hutchison, AT., et al. Effects of intermittent fasting, with and without calorie restriction, on human metabolic health. Obesity Reviews 2016;17:54. | Conference abstract |
|  | Jospe MR, Roy M, Brown RC, Haszard JJ, Meredith-Jones K, Fangupo LJ, et al. Intermittent fasting, Paleolithic, or Mediterranean diets in the real world: exploratory secondary analyses of a weight-loss trial that included choice of diet and exercise. American Journal of Clinical Nutrition. 2020;111(3):503-14. | Type of intervention – 5:2 method |
|  | Kalam F, Gabel K, Cienfuegos S, Ezpeleta M, Pavlou V, Varady KA. Changes in Body Weight in Relation to Appetite During 6 Months of Alternate Day Fasting with a Low Carbohydrate Diet. Current Developments in Nutrition. 2020;4(Supplement_2):1646–. | Conference abstract |
|  | Kalam F, Gabel K, Cienfuegos S, Wiseman E, Ezpeleta M, Steward M, et al. Alternate day fasting combined with a low-carbohydrate diet for weight loss, weight maintenance, and metabolic disease risk reduction. Obesity Science and Practice. 2019;5(6):531-9. | No data for the primary outcome of this review  -Secondary analysis of this study is included in the review (Kalam et al. 2020) |
|  | Kalam F, Gabel K, Wiseman E, Varady K. Alternate Day Fasting Combined with a High Protein/low Carbohydrate Diet: Effect on Body Weight and Metabolic Disease Risk Factors in Obese Adults (P21-018-19). Current developments in nutrition. 2019;3:nzz041. P21-18-19. | Conference abstract |
|  | Kalam F, Kroeger CM, Trepanowski JF, Gabel K, Song JH, Cienfuegos S, et al. Beverage intake during alternate-day fasting: Relationship to energy intake and body weight. Nutrition and health. 2019;25(3):167-71. | No data for the primary outcome of this review |
|  | Keogh JB, Pedersen E, Petersen KS, Clifton PM. Effects of intermittent compared to continuous energy restriction on short-term weight loss and long-term weight loss maintenance. Clinical Obesity. 2014;4(3):150-6. | No data for the primary outcome of this review |
|  | Klempel MC, Kroeger CM, Phillips SA, Varady KA. Alternate Day Fasting With a High Fat and Low Fat Diet: Is Brachial Artery Flow Mediated Dilation Mediated by Adipokine Physiology? : Am Heart Assoc; 2013. | Conference abstract |
|  | Klempel MC, Kroeger CM, Varady KA. Alternate day fasting with a high fat diet: Impact on body weight, body composition, and coronary heart disease risk profile in obese adults. FASEB Journal Conference: Experimental Biology. 2013;27. | No data for the primary outcome of this review  -Secondary analysis of this study is included in the review (Klempel et al. 2013) |
|  | Klempel MC, Kroeger CM, Varady KA. Alternate day fasting increases LDL particle size independently of dietary fat content in obese humans. European journal of clinical nutrition. 2013;67(7):783-5. | No data for the primary outcome of this review |
|  | Kroeger C, Trapanowski J, Barnosky A, Klempel M, Varady K. Effect of 1 year of alternate day fasting versus daily calorie restriction on type 2 diabetes risk. The FASEB Journal. 2015;29:254.1. | Conference abstract |
|  | Kroeger C, Trapanowski J, Klempel M, Bhutani S, Hoddy K, Varady K. Alternate day fasting is effective for weight loss and weight maintenance in obese adults. FASEB Journal Conference: Experimental Biology. 2014;28(1). | Conference abstract |
|  | Kroeger CM, Klempel MC, Bhutani S, Trepanowski JF, Tangney CC, Varady KA. Improvement in coronary heart disease risk factors during an intermittent fasting/calorie restriction regimen: Relationship to adipokine modulations. Nutrition and Metabolism. 2012;9. | Percentage/amount of energy restriction/consumption - energy restricted by 30% of their baseline needs in consecutive fast days |
|  | Kroeger CM, Trepanowski JF, Klempel MC, Barnosky A, Bhutani S, Gabel K, et al. Eating behavior traits of successful weight losers during 12 months of alternate-day fasting: An exploratory analysis of a randomized controlled trial. Nutrition and Health. 2018;24(1):5–10. | Percentage/amount of energy restriction/consumption – There are 2 phase of intervention, the second one consists of 50% restriction of baseline energy needs on fast days. Although the first phase fits with the inclusion criteria, there were no data for primary outcome of this review for the first phase |
|  | Kunduraci YE, Ozbek H. Does the energy restriction intermittent fasting diet alleviate metabolic syndrome biomarkers? A randomized controlled trial. Nutrients. 2020;12(10):1-13. | Type of intervention – time-restricted feeding |
|  | Lin S, Oliveira ML, Gabel K, Kalam F, Cienfuegos S, Ezpeleta M, et al. Does the weight loss efficacy of alternate day fasting differ according to sex and menopausal status? Nutrition, Metabolism and Cardiovascular Diseases. 2020. | No data for the primary outcome of this review |
|  | Maroofi M, Nasrollahzadeh J. Effect of intermittent versus continuous calorie restriction on body weight and cardiometabolic risk markers in subjects with overweight or obesity and mild-to-moderate hypertriglyceridemia: A randomized trial. Lipids in Health and Disease. 2020;19(1). | Percentage/amount of energy restriction/consumption –consumption of 30% of daily calorie requirement on fast days |
|  | Miranda ER, Varady K, Haus JM. Weight loss via alternate day fasting increases circulating endogenous secretory RAGE and is associated with markers of adipocyte health. FASEB Journal Conference: Experimental Biology. 2017;31(1). | Conference abstract |
|  | Oh M, Kim S, An KY, Min J, Yang HI, Lee J, et al. Effects of alternate day calorie restriction and exercise on cardio-metabolic risk factors in overweight and obese adults: an exploratory randomized controlled study. BMC public health. 2018;18(1):1124. | No data for the primary outcome of this review |
|  | Parvaresh A, Razavi R, Abbasi B, Yaghoobloo K, Hassanzadeh A, Mohammadifard N, et al. Modified alternate-day fasting vs. calorie restriction in the treatment of patients with metabolic syndrome: A randomized clinical trial. Complementary Therapies in Medicine. 2019;47. | No data for the primary outcome of this review |
|  | Pedersen E, Jennifer BKJ, Kristina Petersen K, Peter M, Clifton P. Effects of intermittent compared to continuous energy restriction on weight loss and diet quality after one year. Obesity Reviews. 2014;15:142. | Conference abstract |
|  | Razavi R, Parvaresh A, Abbasi B, Yaghoobloo K, Hassanzadeh A, Mohammadifard N, et al. The alternate-day fasting diet is a more effective approach than a calorie restriction diet on weight loss and hs-CRP levels. International Journal for Vitamin and Nutrition Research. 2020. | Paid by the author of this review for full-text.  No data for the primary outcome of this review |
|  | Seighali R, Hojjati Zidashti Z. The effect of regular walking and alternate day fasting on health-related factors in overweight and obese females. Journal of Fasting and Health. 2017;5(1):12-9. | Percentage/amount of energy restriction/consumption –consumption of 30% of daily calorie requirement on fast days |
|  | Sim H, Kim B, Ahn JH, Lee J-C, Lee T-K, Choi JH, et al. Intermittent (alternate-day) fasting for three months increases calbindin D28k immunoreactivity in gerbil hippocampus but does not protect neurons against ischemia-reperfusion injury. 한국실험동물학회 학술발표대회 논문집. 2020:163-. | No data for the primary outcome of this review |
|  | Steger FL, Donnelly JE, Hull HR, Li X, Hu J, Sullivan DK. Intermittent and continuous energy restriction result in similar weight loss, weight loss maintenance, and body composition changes in a 6 month randomized pilot study. Clinical Obesity. 2020. | Percentage/amount of energy restriction/consumption – consumption of 550 to 800 kcal/d on fast days |
|  | Sundfor TM, Tonstad S, Svendsen M. Effects of intermittent versus continuous energy restriction for weight loss on diet quality and eating behavior. A randomized trial. European Journal of Clinical Nutrition. 2019;73(7):1006-14. | Type of intervention – 5:2 method |
|  | Trepanowski J, Kroeger C, Barnosky A, Hoddy K, Varady K. Alternate‐day fasting and daily calorie restriction similarly affect visceral adiposity and circulating inflammatory cytokine concentrations. The FASEB Journal. 2015;29:254.2. | Conference abstract |
|  | Trepanowski JF, Kroeger CM, Barnosky A, Klempel M, Bhutani S, Hoddy KK, et al. Effects of alternate-day fasting or daily calorie restriction on body composition, fat distribution, and circulating adipokines: Secondary analysis of a randomized controlled trial. Clinical Nutrition. 2018(6):1871-8. | No data for the primary outcome of this review |
|  | Trepanowski JF, Kroeger CM, Barnosky A, Klempel MC, Bhutani S, Hoddy KK, et al. Effect of Alternate-Day Fasting on Weight Loss, Weight Maintenance, and Cardioprotection Among Metabolically Healthy Obese Adults: A Randomized Clinical Trial. JAMA Internal Medicine. 2017;177(5):N.PAG-N.PAG. | No data for the primary outcome of this review |
|  | Tripolt N, Stekovic S, Aberer F, Url J, Pferschy P, Obermayer-Pietsch B, et al., editors. Glucose Metabolism after a Fasting Day and after a Day of Eating in healthy Alternate Day Fasting practicing People. WIENER KLINISCHE WOCHENSCHRIFT; 2017: SPRINGER WIEN SACHSENPLATZ 4-6, PO BOX 89, A-1201 WIEN, AUSTRIA. | Full-text not found |
|  | Varady K. Alternate-day fasting. Menopause. 2016;23:1368. | Full-text not found |
|  | Varady KA, Bhutani S, Church EC, Klempel MC. Short-term modified alternate-day fasting: a novel dietary strategy for weight loss and cardioprotection in obese adults. The American journal of clinical nutrition. 2009;90(5):1138-43. | No data for the primary outcome of this review  -Secondary analysis of this study is included in the review (Klempel et al. 2010) |
|  | Varady KA, Bhutani S, Church EC, Klempel MC. Alternate day fasting: A novel dietary strategy for weight loss and cardio-protection in obese adults. FASEB Journal Conference: Experimental Biology. 2010;24. | Conference abstract |
|  | Varady KA, Bhutani S, Klempel M. Weight loss, coronary heart disease risk reduction, and adipokine profile improvement by alternate day fasting. Obesity. 2010;18:S92. | Conference abstract |
|  | Varady KA, Bhutani S, Klempel MC. Effects of alternate day modified fasting on LDL particle size and distribution in obese adults. FASEB Journal Conference: Experimental Biology. 2011;25. | Conference abstract |
|  | Varady KA, Bhutani S, Klempel MC, Kroeger CM. Comparison of effects of diet versus exercise weight loss regimens on LDL and HDL particle size in obese adults. Lipids in Health & Disease. 2011;10:119. | No data for the primary outcome of this review |
|  | Varady KA, Bhutani S, Klempel MC, Kroeger CM, Trepanowski JF, Haus JM, et al. Alternate day fasting for weight loss in normal weight and overweight subjects: a randomized controlled trial. Nutrition Journal. 2013;12(1):146. | BMI range – 50% of the participants were in normal weight range |
|  | Varady KA, Bhutani S, Klempel MC, Lamarche B. Improvements in LDL particle size and distribution by short-term alternate day modified fasting in obese adults. British Journal of Nutrition. 2011;105(4):580-3. | No data for the primary outcome of this review |
|  | Varady KA, Dam VT, Klempel MC, Horne M, Cruz R, Kroeger CM, et al. Effects of weight loss via high fat vs. low fat alternate day fasting diets on free fatty acid profiles. Scientific reports. 2015;5(1):1-7. | No data for the primary outcome of this review |
|  | Varady KA, Gabel K. Dietary adherence and macronutrient intake during 12 months of alternate day fasting. FASEB Journal Conference: Experimental Biology. 2017;31(1). | Conference abstract |
|  | Widhalm K, Poppelmeyer C, Helk O. The Effect of Alternate-Day Fasting (ADF) on Weight Loss, Metabolic Parameters and Psychological Characteristics. Aktuelle Ernahrungsmedizin. 2017;42(3):188-92. | No data for the primary outcome of this review |
|  | Zhang X, Zou Q, Zhao B, Zhang J, Zhao W, Li Y, et al. Effects of alternate-day fasting, time-restricted fasting and intermittent energy restriction DSS-induced on colitis and behavioral disorders. Redox biology. 2020;32:101535. | No data for the primary outcome of this review |
|  | Zheng A, Chen G, Cai K, Liu C. Daily calorie restriction and alternate day fasting for type 2 diabetes prevention. Chinese Journal of Endocrinology and Metabolism. 2016;32(5):433-6. | Full-text not found |
|  | Zuo L, He F, Tinsley GM, Pannell BK, Ward E, Arciero PJ. Comparison of high-protein, intermittent fasting low-calorie diet and heart healthy diet for vascular health of the obese. Frontiers in Physiology. 2016;7. | Duration of intervention – IF for one to two days per month |
|  | 박기덕, editor Effects of 8 weeks of alternate day fasting and combined exercise program on body composition, insulin resistance, and blood lipids in overweight and obese adults: A pilot randomized controlled study2016: ICOCD. | Full-text not found |
